# News and Coming Events

**Published:** 1909-06-26

**Authors:** 


### News and Coming Events.

Fleet-Surgeon A. E. Weightman, L.R.C.P., L.R.C.S., Ed., has been elected a Fellow of the Society of Antiquaries.

Mr. Birrell recently unveiled in the Forbury Gardens at Reading the Memorial Cross in honour of King Henry I. which has been presented to the Corporation of Reading by Dr. Jamieson Boyd Hurry.

The annual meeting of the Friedenheim Hospital (Home of Peace for the Dying) will be held this day, Saturday, June 26, at 3.30 p.m., in the Lecture Hall of the School for the Blind (adjoining Friedenheim), 10 Upper Avenue Road, Swiss Cottage, N.W The chair will be taken by the Mayor of Hampstead, and he will be supported, amongst others, by Mr. John Langton, F.R.C.S. (Chairman of the Council), Dr. A. T. Schofield, and Dr. Percy J. F. Lush.

Prince Alexander of Teck was among the guests at the jubilee dinner of the National Hospital for the Paralysed and Epileptic, Queen Square, Bloomsbury, which was held recently at the Mansion House, under the chairmanship of the Lord Mayor of London. Among those present were Lord Justice Kennedy, the Sheriffs, Sir William Gowers, the Dean of Canterbury, Dr. David Ferrier, Sir Horace Marshall, and Sir Felix Seemon. The Chairman announced on the authority of the Duchess of Albany that the King had consented to open the new building on November 4, while the Princess of Wales would visit the hospital in October to receive purses for the jubilee fund, which aims at raising £50,000. Towards this some £15,000 has already been collected, and during the dinner donations were announced to the amount of £2,719.

Mr. Charles Alfred Ballance, M.V.O., has been appointed a member of the Court of Examiners of the Royal College of Surgeons of England. Mr. Rickman J. Godlee, F.R.C.S., has been appointed to represent the College on the Court of the newly constituted University of Bristol.

The death of Miss Mary Hezmalhalch, of Felliscliffe, Yorks, permits an estate valued at £40,000 to be distributed under the will of her brother, Mr. Joseph Hezmalhalch, who died thirteen years ago. The many charitable legacies included £1,000 each to the Harrogate Bath Hospital and the Harrogate Cottage Hospital; £500 to the Leeds Hospital for Women and Children; £250 each to the Leeds District Nursing Institution, the Ida Convalescent Home, Cookridge, and the Ilkley Bath Hospital. Three-fourths of the residue of the estate were left to the Leeds Infirmary and one-fourth goes to the Leeds Dispensary.

The sixth annual course of instruction in ophthalmology in connection with the University of Oxford will take place this year from July 5 to 17. The first part will consist principally of practical demonstrations in the examination of eye patients, and will include ophthalmoscope work and refraction testing. The second part will be more specialised, and will include lectures by eminent ophthalmic surgeons upon subjects with which, for the most part, their names are identified. The fee for the course is £5 5s. Medical men and students of ophthalmology must secure lodgings for themselves for the first week, but they can be provided with board and residence at Keble College at the rate of 7s. 6d. a day during the second week. All information concerning the course may be obtained from Mr. Robert W. Doyne, M.A., Margaret Ogilvie Reader in Ophthalmology in the University, 30 Cavendish Square, London, W.

The Duchess of Connaught has promised her patronage for the open-air *fête* and bazaar to be held at Isleworth on June 30 and July 1, in aid of the building fund of the new hospital for Hounslow.

Lord Sandhurst, treasurer of St. Bartholomew's Hospital, has received from the Goldsmiths' Company a donation of £500 towards the funds of the hospital in response to an appeal to the City Guilds for assistance in paying off the hospital's existing debt.

The annual tournament of the Medical Golfing Society was held at Burnham Beeches Golf Club on Thursday, June 24. The "Henry Morris" Challenge Cup and the Medical Golfing Society's Gold Medal were competed for, and there were also sweepstakes and other competitions in the afternoon. Any person on the Medical or Dental Registers can join the Society on payment of the annual subscription (4s.), which includes entrance to the tournament. Applications should be sent to Mr. L. Eliot Creasy, 36 Weymouth Street, London, W.

The election of examiners for the ensuing collegiate year of the Royal College of Surgeons of England has resulted as follow: *Primary Fellowship*—Anatomy: F. G. Parsons, Arthur Thomson, C. H. Fagge, James Sherren; Physiology: W. H. Thompson, G. A. Buckmaster, C. F. Myers-Ward, E. W. Wace Carlier; *Conjoint Board*—Elementary Biology: T. W. Shore, J. P. Hill; Anatomy: W. Wright, W. H. Clayton Greene, Arthur Robinson; Physiology: Benjamin Moore, E. H. Starling; Midwifery: W. Rivers Pollock, W. W. Hunt Tate, H. J. M. Playfair, J. H. Targett; Public Health (Part I.): H. R. D. Spitta; (Part II.): R. D. Sweeting.

At the last meeting of the Council of the Pharmaceutical Society of Great Britain, Mr. J. F. Harrington, of Kensington, was elected President in succession to Mr. J. Rymer Young, F.C.S., and Mr. W. L. Currie, of Glasgow, was elected Vice-President. The Hanbury Gold Medal, which is awarded biennially for high excellence in the prosecution or promotion of original research in the chemistry and natural history of drugs, has been awarded to Wilhelm Oswald Alexander Tschirch, Professor of Pharmacognosy and Practical Chemistry at Berne University, who was elected thirteen years ago an honorary member of the Pharmaceutical Society. In 1890 Professor Tschirch was appointed Extraordinary Professor of Pharmacognosy in the Faculty of Medicine at Berne, and succeeded to the chair in 1891.

The Royal Society has approved a scheme for the administration of a Sorby Research Fellowship which has been established in connection with the University of Sheffield. Dr. H. C. Sorby, who was born in Sheffield, left £15,500 for the establishment of a Fellowship for the purpose of carrying out original scientific research. The decision of the Royal Society is to the effect that this Fellowship shall be associated with Sheffield University, so long as, in the opinion of the Society, that University is efficiently equipped in laboratories and appliances. The administration of the fund is vested in a committee consisting of three representatives of the Royal Society, one representative of the Council of Sheffield University, and two representatives of the Senate of that University. The Council of the University has appointed its Vice-Chancellor, Sir Charles Eliot, as its representative, and Professors Hicks and MacDonald have been appointed to represent the Senate.

Lord Rothschild will preside at the first annual meeting of the members of the Association of Subscribers to Charities at the Mansion House on Thursday, July 1, at 3 p.m. Lord Lichfield, Lord Avebury, Lord Brassey, and others will be among the speakers.

The President of the Board of Education has appointed Dr. Ralph H. Crowley, M.D., M.R.C.P., to be an Assistant Medical Officer of the Board. Dr. Crowley, who was for some time honorary physician of the Bradford Royal Infirmary, at present occupies the post of medical superintendent of the Bradford Local Education Authority.

The Council have appointed Miss Kate Brown, M.B., B.S., to be Demonstrator in Anatomy and Curator of the Museum in the London School of Medicine for Women. Lady Northcote will present the prizes and certificates to students for 1908-9 on Friday, July 2, at 4 p.m., when Mrs. Garrett Anderson, M.D., will take the chair.

The first annual dinner of the Society of Tropical Medicine and Hygiene founded two years ago was held on June 18 at the Trocadero Restaurant, London, under the chairmanship of Professor Ronald Ross, C.B., F.R.S. Col. Seely, in proposing the toast of "The Society," referred to the debt which the public owes to the society for its efforts towards abating the diseases of tropical countries. Sir Alfred Jones stated that Liverpool has spent £100,000 on investigations into tropical diseases, and £28,000 in sending out expeditions. Sir Rupert Boyce pointed with satisfaction to the widespread adoption of the principles of tropical sanitation, as first enunciated by Sir Patrick Manson. The Chairman said that the society now numbers nearly 350 members, most of whom are at work in the tropics. He expressed the hope that this country, which has led the way in research in tropical medicine, would now lead the way also in the practical application of those researches. Other toasts and replies were given by Sir Patrick Manson, Sir Archibald Geikie, Mr. Henry Morris, Count Mörner, and Mr. Ramsay Macdonald, M.P. The following telegram was forwarded to Mr. Chamberlain: "The Society of Tropical Medicine thanks you for the great services to tropical medicine rendered by you. Sent at the first banquet of the Society.—Ronald Ross, President."

A complimentary dinner was given at the Grand Hotel, London, last week, to Dr. J. F. W. Tatham on his retirement from the post of Superintendent of Statistics at the General Register Office. Sir Shirley Murphy was in the chair, and he was supported by Sir W. Dunbar (Registrar-General), Mr. Brudenell Carter, and many prominent members of the medical profession and public health service. The arrangements were in the hands of Drs. W. H. Hamer and Herbert Jones. The Chairman submitted the toast of the evening, and spoke in terms of warm appreciation of the guest and his career. He related how Dr. Tatham prepared in 1892 the Manchester life table, the first ever made in relation to any local community. To Dr. Tatham also is due the origination of the present system of weekly and quarterly returns of the Registrar-General giving particulars of notified diseases occurring within the areas of the various medical officers of health. During his 15 years' work at Somerset House he introduced considerable improvements in the classification of the causes of death. Dr. Tatham was a valued member of the Committee on Nomenclature of Diseases of the Royal College of Physicians, and of the Departmental Committee on Physical Deterioration; and he was chairman of the Statistical Committee of the Cancer Research Fund. Dr. Tatham will be succeeded by Dr. Stevenson. The chairman's eulogy was warmly endorsed by the Registrar-General, to whom Dr. Tatham, in responding, paid a generous tribute.

The honorary secretaries of King Edward's Hospital Fund for London have received at the Bank of England, under an order of the High Court, the sum of £7,348 17s. 2d., the residuary estate of the late Mr. Thomas John Bell, bequeathed for charitable purposes.

On the occasion of the Health Congress to be held at Leeds in July the honorary degree of LL.D. will be conferred by the University of that city on the president of the congress (Colonel T. W. Harding) and the honorary degree of D.Sc. on Sir James Crichton-Browne and Major Ronald Ross.

Professor John Chiene, C.B., F.R.S., and F.R.C.S. Ed., LL.D., etc., who has held since 1882 the Professorship of Surgery in Edinburgh University, has applied for permission to retire. The University Court, in granting the application, has agreed that the resignation shall take effect from August 31.

Dr. Paul Langerhans, for 14 years president of the Berlin Municipal Council, died last week in his 90th year. Dr. Langerhans was for many years a member of the Prussian Diet, and in 1881 was elected to the Reichstag, of which he remained a member until 1903. He was an intimate friend of Virchow, and among many civic and political distinctions he received the freedom of the city of Berlin in the Year 1900.

Sir Felix Semon, K.C.V.O., Physician-Extraordinary to the King, is retiring from practice, and the occasion will be marked by a complimentary banquet to be given him by his professional and other friends on Friday, July 2, at the Whitehall Rooms, Hôtel Métropole, at 7.45 p.m. punctually. The organisers of the banquet are anxious to found a lectureship or scholarship in his name, to be a record of his scientific work as one of the founders of the British School of Laryngology. Mr. Henry T. Butlin, F.R.C.S., D.C.L., will preside at the dinner, and all communications with regard to the Testimonial Fund should be addressed to the hon. secretaries: Mr. Alfred Mond, M.P., 35 Lowndes Square, S.W.; Dr. P. Watson Williams, 4 Clifton Park, Bristol; Mr. H. J. Davis, 8 Portman Street, London, W. The price of tickets for the banquet is £1 1s. inclusive, and ladies as well as gentlemen are invited. Mr. H. J. Davis is the acting hon. secretary to the Dinner Committee, and it is requested that applications for tickets for the banquet should be addressed to him. Cheques for the tickets should be sent with applications.

